# Utilization and Prognosis of Cardiac Device Implantation in AL Versus ATTR Amyloidosis

**DOI:** 10.1111/pace.70180

**Published:** 2026-02-25

**Authors:** Bilal Hussain, Sanchit Duhan, Khawaja Hassan Akhtar, Amith Reddy Seri, Krittapoom Akrawinthawong, Janardhana Gorthi, Bijeta Keisham, Tarun Dasari

**Affiliations:** ^1^ Division of Cardiology University of Cincinnati Cincinnati Ohio USA; ^2^ Division of Cardiology Carle Foundation Hospital Urbana Illinois USA; ^3^ Division of Cardiology University of Oklahoma Health Sciences Center Oklahoma City Oklahoma USA; ^4^ Division of Internal Medicine McLaren Healthcare Flint Michigan USA; ^5^ Division of Advanced Heart Failure Transplant Cardiology Houston Methodist DeBakey Heart & Vascular Center Houston Texas USA; ^6^ Division of Internal Medicine Weifang Medical University Weifang China

**Keywords:** amyloidosis, ATTR amyloidosis, AL amyloidosis

## Abstract

**Introduction:**

Cardiac amyloidosis can cause congestive heart failure, arrhythmias, and heart blocks, which frequently require cardiac device implantation (CDI). However, the differences between light chain (AL) amyloidosis and transthyretin (ATTR) amyloidosis CDI requirements are unknown.

**Methods:**

A retrospective analysis was conducted using the National Inpatient Sample 2017–2020 with respective ICD‐10 codes. Weighted multivariate regression was used to analyze in‐hospital mortality by adjusting for confounders.

**Results:**

Among 1,008,010 patients hospitalized for CDI, 160 patients had underlying AL‐AMD, while 200 patients had underlying ATTR‐AMD. Patients undergoing CDI with underlying ATTR‐AMD had a higher mean age (76.7 vs. 68.4 years, *p* = 0.001), and a higher male proportion (80% vs. 46.8%, *p* = 0.005) as compared to those with AL‐AMD. ATTR‐AMD had a higher CDI rate than AL‐AMD (1.4% vs. 8.3%). The predominant device implanted in both AL‐AMD and ATTR‐AMD was a permanent pacemaker (PPM) (0.8% vs. 5.7%). Underlying AL‐AMD was associated with higher in‐hospital mortality in patients undergoing PPM (OR 5.1, CI 1.3–19, *p* = 0.01), and implantable‐cardioverter defibrillator (ICD) implantation (OR 21.5, CI 4.6–99, *p *< 0.001). Patients undergoing CDI with underlying AL‐AMD had a higher mean length of stay.

**Conclusion:**

ATTR‐AMD has a higher CDI rate as compared to AL‐AMD. Underlying AL‐AMD is associated with higher in‐hospital mortality in patients undergoing PP and ICD implantation.

**Abbreviations**
ATTRv Amyloidosis: hereditary transthyretin amyloidosis
AL‐AMD: light chain amyloidosis
ATTR‐AMD: transthyretin amyloidosis
ATTRwt Amyloidosis: wild‐type transthyretin amyloidosis
CDI: cardiac device implantation
CRT‐P/D: cardiac resynchronization therapy‐pacemaker/defibrillator
ICD: implantable‐cardioverter defibrillator
PPM: permanent pacemaker

## 1 Introduction

Amyloidosis is a rare, debilitating disease caused by protein misfolding, leading to amyloid fibril aggregation and organ deposition. Amyloid fibrils can deposit in the myocardium's interstitial spaces, leading to infiltrative heart disease [1]. The morbidity and mortality associated with amyloidosis are mainly seen with the involvement of the heart, kidney, liver, and autonomic nervous system. The two most common types of amyloidosis affecting the heart include light chain (AL) amyloidosis (AL‐AMD) and transthyretin amyloidosis (ATTR‐AMD) [2, 3]. AL amyloidosis results from the deposition of immunoglobulin light chains produced by plasma cell dyscrasia, while ATTR amyloidosis results from the deposition of misfolded transthyretin protein [4].

AL‐AMD patients have a median survival time of <6 months [5], but it has nearly doubled due to therapeutic advances with stem cell transplantation and chemo/immunotherapy. ATTR‐AMD, with a median survival of 75 months, has a more favorable prognosis than AL‐AMD. The infiltrative process of the amyloid in the heart can cause progressive muscle dysfunction, causing heart failure and affecting the conduction system, resulting in bradyarrhythmias [6]. Recent data indicate the utility of implantable‐cardioverter defibrillators (ICD) in managing ventricular arrhythmias and preventing sudden cardiac death [7]. Additionally, permanent pacemakers (PPM) are indicated for conduction disorders in amyloidosis [8]. There is a lack of large‐scale data on the characteristics, in‐hospital mortality, and utilization of cardiac devices in AL and ATTR amyloidosis. We aimed to study the current real‐world scenario in applying these treatment modalities.

## 2 Methods

### 2.1 Study Data and Population

The study was conducted using the National Inpatient Sample (NIS) from January 1, 2017, to December 31, 2020, as International Classification of Diseases, Tenth Revision, Clinical Modification (ICD‐10‐CM) codes for AL‐AMD and ATTR‐AMD were introduced in the third quarter of 2016. NIS is the largest publicly available administrative claims database, which contains data for around 35 million weighted hospitalizations nationally. Patients hospitalized for cardiac device implantation (CDI) (*n* = 1,008,010) were identified using respective ICD‐10 procedure codes. The cardiac devices studied include PPM, ICD, and cardiac resynchronization therapy‐pacemaker/defibrillator (CRT‐P/D). These patients with CDI were divided into two cohorts: AL‐AMD and ATTR‐AMD, which were compared for outcomes.

### 2.2 Patient Characteristics and Study End Points

For baseline characteristics, we compared CDI patients with underlying AL‐AMD and ATTR‐AMD in terms of age, gender, ethnicity, hospital characteristics, Charlson Comorbidity Index (CCI), and baseline comorbidities. Furthermore, we reported the prevalence of various cardiac devices in patients with underlying AL‐AMD and ATTR‐AMD. The primary outcome was in‐hospital mortality in patients undergoing CDI with underlying AL‐AMD versus ATTR‐AMD. Secondary outcome was mean hospitalization costs (adjusted for inflation according to 2022) and mean length of stay in patients hospitalized for CDI with underlying AL‐AMD versus ATTR‐AMD.

### 2.3 Statistical Analysis

Descriptive statistics were utilized to summarize frequencies and proportions for categorical variables, while continuous variables were reported as means. Pearson chi‐square test (*χ* ^2^) and Fisher's exact test were used to compare baseline characteristics for categorical variables. Univariate logistic regression model was used to report the effect of underlying AL‐AMD versus ATTR‐AMD in patients hospitalized for CDI. Multivariate logistic regression was implemented to calculate the adjusted effect by accounting for age, gender, ethnicity, median household income, insurance, hospital bed‐size, teaching status, location, and CCI. Linear regression models were used to compare continuous outcomes of mean length of stay and total charges. All analyses were performed using Stata Statistical Software Version 17.

## 3 Results

### 3.1 Baseline Characteristics of CDI With Underlying AL vs. ATTR‐AMD

Baseline characteristics for patients undergoing CDI with underlying AL‐AMD and ATTR‐AMD are compared in Table 1. Patients hospitalized for CDI with underlying ATTR‐AMD were older than those with AL‐AMD (76.7 vs. 68.4 years, *p* = 0.001), and had a higher male population (80% vs. 46.8%, *p* < 0.01). In terms of comorbidities, patients with ATTR‐AMD and CDI had a higher obese population (22.5% vs. 3.1%, *p* = 0.04). Other comorbidities were not significantly different between AL‐AMD and ATTR‐AMD patients undergoing CDI (Table 1). Baseline characteristics of patients undergoing ICD, PPM, and CRT patients with AL‐AMD and ATTR‐AMD are compared in Tables S1–S2, with a higher mean age of CRT patients with AL‐AMD (77 years).

**TABLE 1:** Baseline characteristics for cardiac device implantation with AL‐AMD versus ATTR‐AMD.

	CDI (*n* = 1,008,010)				
	With AL‐AMD (*n* = 160)		With ATTR‐AMD (*n* = 200)		
Patient characteristic	Number of patients (*n*)	Proportion (%)	Number of patients (*n*)	Proportion (%)	*p* value
Age					
Mean age (years)	68.4		76.7		0.001
Gender					
Male	75	46.8	160	80	0.005
Female	85	53.2	40	20	
Ethnicity					
White	80	51.6	130	66.7	0.08
African‐American	35	22.6	50	25.6	
Others	45	25.8	20	7.7	
Co‐morbidities					
Coronary artery disease	60	37.5	105	52.5	0.19
Congestive heart failure	135	84.4	175	87.5	0.7
Arrhythmia	150	93.8	200	100	0.6
Hypertension	125	78.1	170	85	0.45
Diabetes	35	21.9	50	25	0.76
Hyperlipidemia	105	65.6	130	65	0.95
Peripheral artery disease	< 11	3.1	—	—	—
Chronic pulmonary disease	30	18.8	45	22.5	0.69
Liver disease	20	12.5	< 11	5	0.26
Kidney disease	125	78.1	140	70	0.4
Anemia	70	43.8	60	30	0.2
Obesity	< 11	3.1	45	22.5	0.04
Smoking	< 11	3.1	< 11	2.5	0.87
Charlson Comorbidity Index					
0	—	—	< 11	2.5	0.1
1	15	9.4	30	15	
2	< 11	6.3	30	15	
≥ 3	135	84.4	135	67.5	
Median household income					
$1–$49,999	30	19.4	60	30	0.9
$50,000–$85,999	95	58	90	45	
> $86,000	35	22.6	50	25	
Insurance					
Medicare	90	56.3	135	67.5	0.07
Medicaid	< 11	3.1	15	7.5	
Private Insurance	50	31.3	50	25	
Others	15	9.3	0	—	
Hospital characteristics					
Hospital bed‐size					
Small	< 11	6.3	25	12.5	0.6
Medium	35	21.9	35	17.5	
Large	115	71.8	140	70	
Location/teaching status					
Rural	—	—	—	—	0.56
Urban non‐teaching	< 11	6.3	20	10	
Urban teaching	150	93.7	180	90	
Region					
Northeast	30	18.8	65	32.5	0.03
Midwest	40	25	70	35	
South	45	28.1	40	20	
West	45	28.1	25	12.5	
Ownership					
Government	< 11	6.3	25	12.5	0.4
Private	150	93.7	175	87.5	

### 3.2 Prevalence of CDI in AL‐AMD and ATTR‐AMD

**FIGURE 1:**
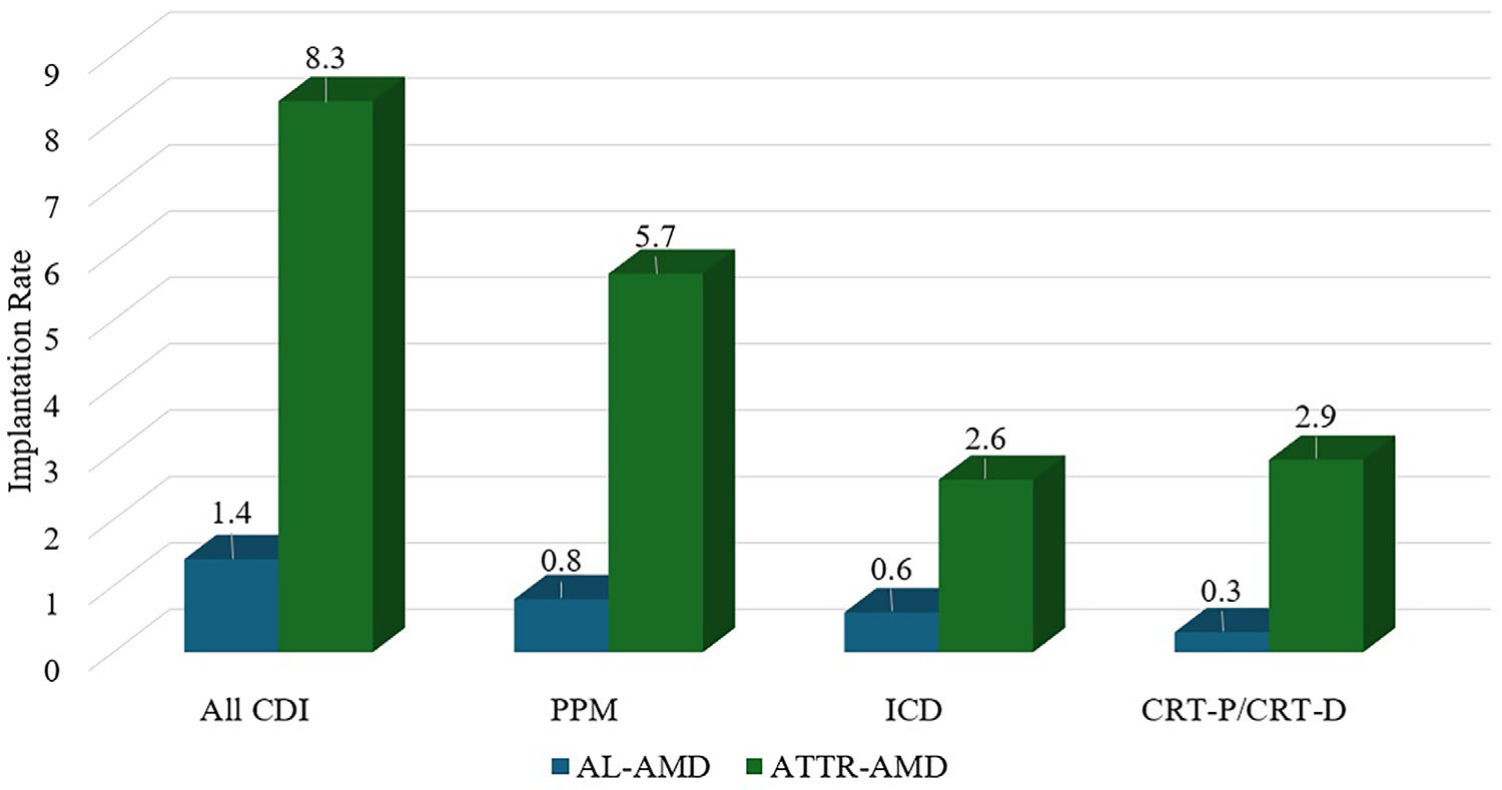
Prevalence of cardiac device implantation in AL‐AMD and ATTR‐AMD. [Colour figure can be viewed at wileyonlinelibrary.com]

Among 11,220 admitted with AL‐AMD, 160 patients underwent CDI (prevalence 1.4%) (Figure 1, Table 2). The majority of AL‐AMD patients were admitted for undergoing PPM (0.8%), followed by ICD (0.6%), and CRT‐P/D (0.3%) placement. While, prevalence of CDI was much higher among the patients with ATTR‐AMD, that is, 8.3%. The predominant device placed in ATTR‐AMD was PPM (5.7%), followed by CRT‐P/D (2.9%), and ICD (2.6%).

**TABLE 2:** Prevalence of cardiac device implantation in AL‐AMD and ATTR‐AMD.

	AL‐AMD (*n* = 11,220)			ATTR‐AMD (*n* = 2455)		
Cardiac device	Number of patients (*n*)	Proportion (%)	*p* value	Number of patients (*n*)	Proportion (%)	*p* value
All cardiac devices	160	1.43	< 0.001	200	8.2	< 0.001
PPM	95	0.8	< 0.001	140	5.7	< 0.001
ICD	70	0.6	< 0.001	65	2.6	< 0.001
CRT‐P/CRT‐D	30	0.3	< 0.001	70	2.9	< 0.001

### 3.3 In‐Hospital Mortality Outcomes of Underlying AL‐AMD vs. ATTR‐AMD in CDI

**FIGURE 2:**
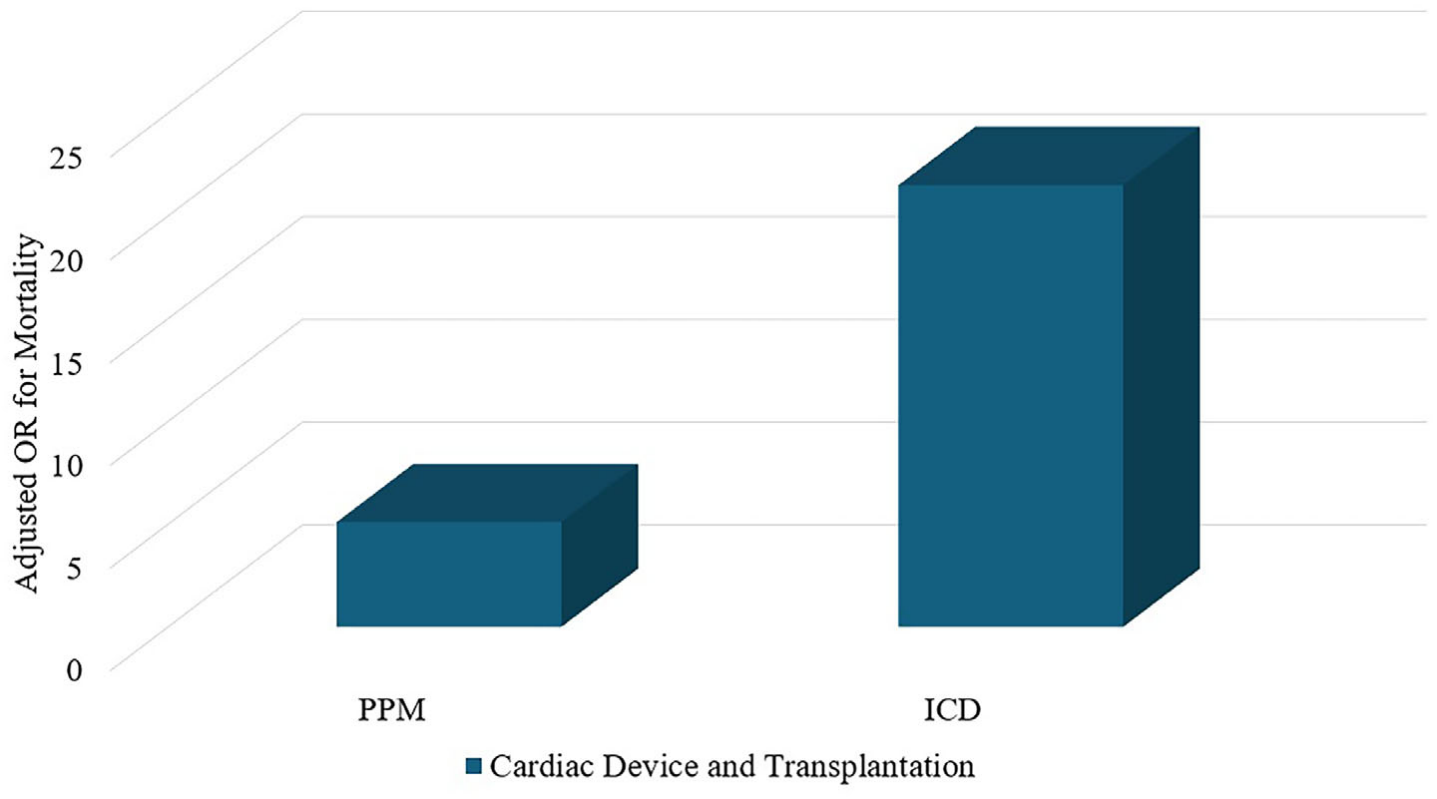
Primary outcome: Effect of underlying AL‐AMD in CDI. [Colour figure can be viewed at wileyonlinelibrary.com]

Figure 2 and Table 3 demonstrate the effect of underlying AL‐AMD on in‐hospital mortality in patients hospitalized for CDI. Underlying AL‐AMD was associated with over five times higher in‐hospital mortality in patients admitted for PPM implantation (OR 5.1, CI 1.3–19, *p* = 0.01), and over 21 higher in‐hospital mortality in patients admitted for ICD implantation (OR 21.5, CI 4.6–99, *p* < 0.001). While AL‐AMD was not associated with in‐hospital mortality in patients undergoing CRT‐P/D placement. Furthermore, no mortality was observed in patients undergoing CDI with underlying ATTR‐AMD.

**TABLE 3:** Primary outcome: effect of underlying AL‐AMD in CDI.

	AL‐AMD *n* = 11,795	Univariate logistic regression		Multivariate logistic regression	
Cardiac device/transplantation	Number of deaths (mortality rate)	OR (95% CI)	*p* value	OR (95% CI)	*p* value
PPM	15 (15.8)	10.8 (3.2–37)	< 0.001	5.1 (1.3–19)	0.01
ICD	< 11 (14.3)	16.7 (3.7–74)	< 0.001	21.5 (4.6–99)	< 0.001
CRT‐P/CRT‐D	—	—	—	—	—

### 3.4 Length of Stay and Hospitalization Costs in CDI With AL‐AMD vs. ATTR‐AMD

Patients undergoing CDI with underlying AL‐AMD had a higher mean length of stay as compared to those without AL‐AMD (12.3 vs. 6.6 days, *p* = 0.01). While AL‐AMD was not associated with higher mean adjusted total charges in CDI ($326,480.3 vs. $193,722.3; *p* = 0.12) (Table S3). Additionally, ATTR‐AMD had no association with mean length of stay (8.03 vs. 6.6 days, *p* = 0.32), or mean adjusted total charges ($181,633.4 vs. $193,746; *p* = 0.34) (Table S4).

## 4 Discussion

The key findings in this NIS database analysis of CDI in amyloidosis patients include (1) Patients with ATTR‐AMD and CDI had a higher mean age and were predominantly males, (2) ATTR‐AMD had a higher CDI rate than AL‐AMD, (3) Underlying AL‐AMD is associated with higher in‐hospital mortality in patients undergoing PPM and ICD implantation.

### 4.1 Baseline Characteristics

AL amyloidosis is caused by clonal plasma cell disorders leading to excess secretion of immunoglobulin fragments, usually immunoglobulin light chains, and leading to amyloid deposition [9]. It is a life‐threatening condition characterized by multi‐organ involvement and rapid loss of organ function compared to other amyloidosis types. This stems from the increased toxicity of amyloidogenic light chains at lower concentrations than different types of amyloid fibrils, which has been well demonstrated in cardiomyocytes [10, 11, 12, 13, 14]. The heart is the most involved organ (70%–80% of patients), causing heart failure [15]. AL‐AMD is diagnosed at a mean age of 63, with 90% of patients ≥ 50 years old [12]. ATTR amyloidosis has two variants: wild‐type (ATTRwt) and hereditary (ATTRv) amyloidosis. ATTRwt is caused by normal protein transthyretin (TTR) and mainly affects older individuals with a median 75‐year age at diagnosis and > 90% reported cases in males to date [16]. Almost two‐third patients present with heart failure at diagnosis [16]. On the other hand, ATTRv amyloidosis is caused by a mutated TTR protein and a 39‐year median age of disease onset, with cardiac manifestations seen in 40% of cases [17, 18]. This study is limited in identifying the types of ATTR amyloidosis. However, since the cohort was focused on CDIs, it is safe to say that more ATTRwt cases were included compared to ATTRv, given the higher likelihood of cardiac involvement in the ATTRwt subtype.

The prevalence of different amyloidosis subtypes and their propensity to cause cardiac symptoms correlates well with the demographics of CDI seen in this study. Younger age in AL amyloidosis patients with a more aggressive disease pathology than ATTRwt amyloidosis explains the higher mean age for CDI in ATTR amyloidosis. Also, a higher probability of male patients in the ATTR amyloidosis group correlated with higher males requiring CDI in this group. AL amyloidosis commonly affects kidneys (50%–60% of patients) and also causes peripheral neuropathy, macroglossia, dysphagia, and easy bruising [15]. Hepatic and pulmonary amyloidosis, although associated with AL amyloidosis, are rarely seen [19, 20]. However, ATTRwt predominantly presents with cardiac complications such as heart failure, atrial flutter/fibrillation, and conduction abnormalities [16, 21]. This study's higher systemic co‐morbidities in the AL amyloidosis group and cardiac co‐morbidities in the ATTR amyloidosis group parallel the previously observed data, despite the CDI in the cohorts.

### 4.2 Permanent Pacemaker and Cardiac Resynchronization Therapy

In addition to restrictive cardiomyopathy, patients with cardiac amyloidosis present with arrhythmia and conduction abnormalities, which include atrial fibrillation, sinus node dysfunction, and conduction blocks [8]. Pacemakers are frequently indicated in these patients, mainly in ATTR amyloidosis [8]. Higher prevalence of PPM implantation was observed in ATTR as compared to AL‐AMD due to predominant cardiac involvement (5.7% vs. 0.8%). The role of CRT is well established in patients with heart failure with reduced ejection fraction (HFrEF), but very limited data exist evaluating efficacy of CRT in cardiac amyloidosis patients. Patients with amyloidosis are often PPM dependent, and there is a concern for dyssynchrony due to chronic RV pacing, which favors the implantation of CRT. Donellan et al. reported that higher right ventricular (RV) pacing burden is associated with CHF and deleterious remodeling in cardiac amyloidosis, while biventricular (BiV) pacing is associated with improved NYHA CHF class, LVEF, and mitral regurgitation, and recommended considering CRT as compared to PPM in amyloidosis patients requiring pacing [22]. Another study observed improved survival and heart failure symptoms in patients with CRT and ATTR‐AMD [23], whereas a study by Fischer et al. [24] reported lower rates of CRT response and worse CV outcomes in patients with cardiac amyloidosis after CRT implantation. Our study showed that underlying AL‐AMD was associated with worse prognosis in patients undergoing PPM implantation, while no association was seen for CRT. Patients with AL‐AMD have a hematologic malignancy with multi‐organ involvement, and the prognosis depends on the degree of cardiac involvement.

### 4.3 Implantable‐Cardioverter Defibrillator

Sudden cardiac death ranks among the most common forms of death in patients with CA, but results from pulseless electric activity (PEA) or non‐defibrillable rhythms much more often than tachyarrhythmias [25]. Risk of electromechanical dissociation, defibrillation thresholds, and complication rates are higher in these patients. The guidelines regarding ICD placement in amyloidosis are unclear, as the studies evaluating their use have contradictory results [7, 26]. Due to insufficient data, the European Society of Cardiology 2015 guidelines do not recommend preventive ICD implantation in cardiac amyloidosis patients [27]. Heart Rhythm Society 2019 guidelines suggest that a prophylactic ICD may be considered in AL‐AMD patients with non‐sustained ventricular tachycardia and expected survival of more than 1 year (Class IIb recommendation) [28]. Several studies have identified successful ICD therapy, which was not associated with any survival benefit [7, 26, 29, 30, 31, 32]. However, these studies assessing the ICD implantation outcomes do not differentiate between AL and ATTR‐AMD, which majorly limits the assessment of their results, given the markedly different clinical presentation, natural history, progression, and treatment of the two types. Electrical abnormalities such as epicardial conduction and repolarization abnormalities are more commonly seen in AL‐AMD than ATTR‐AMD [33]. HV interval, which represents the conduction time from the proximal His bundle to the ventricular myocardium, is more commonly prolonged in ATTR‐AMD [6]. A hallmark feature of ATTRv amyloidosis is autonomic dysfunction, but the cardiac manifestations leading to sustained arrhythmias are unclear [34]. The conduction abnormalities and autonomic dysfunction possibly explain the higher ICD implantation in ATTR‐AMD patients seen in this study. Patients with AL‐AMD have a poor long‐term prognosis with a median survival time < 6 months [5], which correlates with higher in‐hospital mortality in patients undergoing ICD implantation with underlying AL‐AMD.

### 4.4 Limitations

Some significant limitations need to be considered while assessing the results of this study. The NIS database does not allow differentiation between ATTRwt and ATTRv amyloidosis. The retrospective design of the study has inherent tendencies of selection bias. The large database is vulnerable to limitations of under‐ and over‐coding. Given the cross‐sectional nature of the data, the hospital courses and the trajectory of the events leading to the outcomes cannot be studied. This follow‐up data is unavailable after discharge, and long‐term outcomes cannot be evaluated. Despite these limitations, analyzing an extensive database is essential for gaining insight into real‐world practices and hypothesis building. This study's results aim to help clinicians make informed decisions in the management of AMD patients.

## 5 Conclusion

In conclusion, the use of CDI is higher in ATTR‐AMD patients than in AL‐AMD. The in‐hospital mortality is higher in patients undergoing PPM implantation and ICD implantation with underlying AL‐AMD; however, the association of CDI with the progression of the underlying disease and its impact on prolonging survival is unclear. Prospective studies assessing the proportionally adjusted in‐hospital mortality rates in AL and ATTR‐AMD patients with CDI would help determine their efficacy and benefits.

## Author Contributions

**Bilal Hussain**: writing – original draft, review and editing, formal analysis. **Sanchit Duhan**: writing – original draft, review and editing, visualization. **Khawaja Hassan Akhtar**: writing – review and editing, supervision, validation. **Amith Reddy Seri**: data curation, validation. **Krittapoom Akrawinthawong**: writing – review and editing, supervision, validation. **Janardhana Gorthi**: writing – review and editing, supervision, validation. **Bijeta Keisham**: data curation, validation. **Tarun Dasari**: writing – review and editing, supervision, validation.

## Funding

The authors have nothing to report.

## Conflicts of Interest

The authors declare no conflicts of interest.

## Supporting information




**Table S1**: Baseline Characteristics for Different Cardiac Device Implantations with AL‐AMD.
**Table S2**: Baseline Characteristics for Different Cardiac Device Implantations with ATTR‐AMD.
**Table S3**: Secondary Outcome: Adjusted Total Charges and Length of Stay For CDI with Underlying AL‐AMD.
**Table S4**: Secondary Outcome: Adjusted Total Charges and Length of Stay For CDI with Underlying ATTR‐AMD.
