# Supplementary material for: Utilization and Prognosis of Cardiac Device Implantation in AL Versus ATTR Amyloidosis
Source: Pacing Clin Electrophysiol. 2026 Feb 25;49(7):954–61. doi: 10.1111/pace.70180 (PMC13331598; doi:10.1111/pace.70180)
Supplement: Supplementary file 1 — Table S1: Baseline Characteristics for Different Cardiac Device Implantations with AL‐AMD. Table S2: Baseline Characteristics for Different Cardiac Device Implantations with ATTR‐AMD. Table S3: Secondary Outcome: Adjusted Total Charges and Length of Stay For CDI with Underlying AL‐AMD. Table S4: Secondary Outcome: Adjusted Total Charges and Length of Stay For CDI with Underlying ATTR‐AMD. [file PACE-49-954-s001.docx]

**Supplementary Tables**

**Table S1: Baseline Characteristics for Different Cardiac Device Implantations with AL-AMD**

| Characteristic | ICD & AL-AMD | PPM & AL-AMD | CRT & AL-AMD | p Value |
| --- | --- | --- | --- | --- |
| Age |  |  |  |  |
| Mean Age (years) | 57.9 +2 | 75.6 +2 | 77 +4.7 | <0.001 |
| Gender |  |  |  |  |
| Male | 30 (46) | 40 (42.1) | 20 (66.7) | 0.9 |
| Female | 35 (54) | 55 (57.9) | <11 (33.3) |  |
| Ethnicity |  |  |  |  |
| White | 35 (53.9) | 45 (47.4) | <11 (33.3) | 0.98 |
| African-American | 15 (23) | 25 (26.3) | 15 (50) |  |
| Others | 15 (23.1) | 25 (26.3) | <11 (16.7) |  |

**Table S2: Baseline Characteristics for Different Cardiac Device Implantations with ATTR-AMD**

| Characteristic | ICD & ATTR-AMD | PPM & ATTR-AMD | CRT & ATTR-AMD | p Value |
| --- | --- | --- | --- | --- |
| Age |  |  |  |  |
| Mean Age (years) | 73.5 +2.9 | 78.4 +1.3 | 78 +1.5 | 0.12 |
| Gender |  |  |  |  |
| Male | 50 (77) | 110 (82.1) | 60 (93) | 0.2 |
| Female | 15 (23) | 25 (18) | <11 (7) |  |
| Ethnicity |  |  |  |  |
| White | 25 (38.5) | 105 (77.8) | 50 (77) | 0.06 |
| African-American | 40 (61.5) | 15 (11.1) | 15 (23) |  |
| Others | - | 15 (11.1) | - |  |

**Table S3: Secondary Outcome: Adjusted Total Charges and Length of Stay For CDI with Underlying AL-AMD**

| Outcome | CDI with AL-AMD | | CDI without AL-AMD | | p Value |
| --- | --- | --- | --- | --- | --- |
|  | **Value** | **Standard Error** | **Value** | **Standard Error** |  |
| Mean Adjusted Total Charges ($) | 326480.3 | 57470 | 193722.3 | 1546.6 | 0.14 |
| Mean Length of Stay (Days) | 12.28 | 2.04 | 6.6 | 0.03 | 0.01 |

**Table S4: Secondary Outcome: Adjusted Total Charges and Length of Stay For CDI with Underlying ATTR-AMD**

| Outcome | CDI with ATTR-AMD | | CDI without ATTR-AMD | | p Value |
| --- | --- | --- | --- | --- | --- |
|  | **Value** | **Standard Error** | **Value** | **Standard Error** |  |
| Mean Adjusted Total Charges ($) | 181633.4 | 17632.3 | 193746 | 1546.95 | 0.34 |
| Mean Length of Stay (Days) | 8.03 | 0.86 | 6.6 | 0.03 | 0.32 |
